# Improved treatment of vulvovaginal candidiasis with Clotrimazole plus probiotic Lacidophilin Vaginal Capsules: A prospective, real-world study

**DOI:** 10.1097/MD.0000000000032664

**Published:** 2023-01-06

**Authors:** Xianling Zeng, Ruifang An, Han Li, Yafei Zhang

**Affiliations:** a From the Department of Gynecology, The First Affiliated Hospital of Zhengzhou University, Zhengzhou, Henan, China; b From the Department of Obstetrics and Gynecology, The First Affiliated Hospital of Xi’an Jiaotong University, Xi’an, Shaanxi, China; c From the Department of Hepatobiliary and Pancreatic surgery, The First Affiliated Hospital of Zhengzhou University, Zhengzhou, Henan, China.

**Keywords:** Lactobacillus, probiotic, treatment, vaginal microbiota, vulvovaginal candidiasis

## Abstract

Clotrimazole has long been used to treat vulvovaginal candidiasis (VVC), yet the antibiotic resistance, adverse effects and recurrences still bring about a great challenge for the clinicians. To explore the effect of probiotic Lacidophilin Vaginal Capsules plus Clotrimazole Vaginal Tablets (500mg) in the treatment of uncomplicated VVC, a self-controlled real-world study was conducted.

**Methods:**

Twenty-seven women with a normal vaginal flora and 15 women with uncomplicated VVC were recruited. The patients were treated with the single dose of Clotrimazole Vaginal Tablets (500mg) supplemented with 2 Lacidophilin Vaginal Capsules for the following 7 days. The patients were prospectively examined 4 times and the time points were at m0 (the first visit), m1 (8–10 days after the first visit), m2 (30 days after the second visit) and m3 (30 days after the third visit). However, women in the healthy normal control group were examined just once at the first visit. The obtained vaginal secretions were examined by high-throughput sequencing.

**Results:**

The mean age in healthy control group and case group was 28.63 ± 5.40y and 27.67 ± 3.33y, respectively. Finally, 46.67% (7/15) of patients were cured at the second visit, 61.54% (8/13) were cured at the third visit and eventually 72.73% (8/11) were cured. A total of 81 samples were sequenced, generating 1668 operation taxonomy units among all the samples. The bacterial composition of women in the healthy control group was exceedingly abundant and dominated by Lactobacillus, especially by Lactobacillus. crispatus, and followed by Lactobacillus. iners, Lactobacillus. jensenii and Gardneralla. On the contrary, the bacterial composition of women with VVC was relatively few and dominated by Lactobacillus. iners. During the process of treatment, the bacterial abundance of VVC patients was increased gradually. At the final visit, the abundance of vaginal flora was augmented further with the dominant bacteria being Lactobacillus. crispatus, followed by Lactobacillus. iners.

**Conclusion:**

Clotrimazole Vaginal Tablets plus probiotic Lacidophilin Vaginal Capsules could improve the effect in treating uncomplicated VVC. This improved effect was achieved perhaps through improving the composition of vaginal flora and restoring vaginal microecology.

## 1. Introduction

Vulvovaginal candidiasis (VVC) is a common and widespread vaginal infectious disease.^[1]^ The typical clinical presentation of VVC includes white cheese-like discharge with vulvar erythema, edema, ulcers and fissures. The opportunistic fungal pathogen, *Candida albicans* (*C. albicans*), is responsible for 85% to 90% of VVC cases. The occurrence of VVC involves a range of risk factors, such as uncontrolled diabetes, long-term or repeated exposure to antibiotics, and unhealthy lifestyle habits.^[2]^ Nearly 75% of women of childbearing age will suffer from VVC at least once in their lifetime, and 40% to 50% of them will experience a recurrence of VVC. Such a high incidence rate, combined with the enormous medical costs, highlights the urgency of understanding the pathogenesis of VVC. Therefore, there is an urgent need to develop innovative strategies to prevent and control it.

The recommended standard therapy for vaginal *C. albicans* infection is antifungal therapy, including oral or intravaginal azole or triazole.^[3]^ Although Clotrimazole has long been used to treat VVC, antibiotic resistance, adverse effects and relapse continue to pose significant challenges to clinicians. To overcome these difficulties, researchers have recently turned their attention to the complementary role of probiotics, such as *Lactobacilli*, in VVC treatment. The concept of combining antibiotics and probiotics stems from a traditional theoretical basis that a normal vaginal microbial community plays an important role in the prevention of yeast infections. On the other hand, this notion is reinforced by the many observations that long-term antibiotic use can lead to yeast infections. However, the relationship between vaginal microflora and Candida-induced VVC is still poorly understood.

Currently, there has emerged growing interest in the therapeutic use of probiotics.^[4]^ A probiotic is a product that contains a certain number of microorganisms that can influence the microbiota of a certain compartment of a host or exert some beneficial effect in that host. Studies have found that probiotic strains possess the ability to prevent infectious diarrhea and relieve ulcerative colitis and pouchitis.^[5–7]^

*Lactobacillus* species can produce both lactic and hydrogen peroxide (H_2_O_2_). Also, *Lactobacillus* has the ability to maintain vaginal pH not exceeding 4.5 and inhibit the growth of pathogenic microorganisms and *C. albicans*. Therefore, they are considered a protective factor against VVC. They are now being used to restore the physiological vaginal microbiota in women suffering from vaginal dysbiosis.^[1,8–10]^ Studies have shown that probiotics have the potential to improve the cure rate of bacterial vaginosis (BV) ^[11]^ and can improve the relative abundance of vaginal *Lactobacilli* in BV patients. *Lactobacillus plantarum* P17630 was shown to be effective in preventing VVC recurrence.^[12]^ In addition, *L. plantarum* 59 and *L. fermentum* 137 are interesting probiotic candidates for the prevention or treatment of VVC.^[13]^ Moreover, *L. crispatus* and *L. delbrueckii* showed potential for adjuvant biotherapeutic effect in treating VVC in a Sprague-Dawley rat model.^[14]^ In *vitro* experiments have shown that the probiotics *Lactobacillus rhamnosus* GR-1 and *Lactobacillus reuteri* RC-14 can effectively hinder the growth of *C. albicans* and even kill them.^[15]^ However, some studies have found no significant effect of probiotics on the cure rate of VVC.^[1]^ Besides, Lopes et al found that *L. delbrueckii* showed no antimicrobial activity against pathogens.^[16]^ There is considerable heterogeneity between the results of various studies.

The impractical and expensive “cultivation-dependent” method, traditionally used to analyze large numbers of samples are further limited by their dependence on certain selective media. Moreover, the fact that some bacteria are difficult to culture could not be conquered recently. Emerging sequencing technologies based on 16S rRNA gene analysis can overcome these limitations and are therefore widely used to explore microbial diversity, making high-throughput sample analysis possible. Importantly, this approach can provide precise details of the detected samples. In the present study, we used this emerging approach to characterize the composition and structure of the vaginal flora in healthy women and women with VVC to explore the effect of probiotic Lacidophilin Vaginal Capsules plus Clotrimazole Vaginal Tablets (250 mg per tablet) on improving the treatment of uncomplicated VVC.

## 2. Materials and methods

### 2.1. Subjects

This prospective study was conducted at the Gynecology Outpatient Clinic of the First Affiliated Hospital of Xi’an Jiaotong University from March 2018 to December 2018 under real-world conditions. Eligible participants needed to satisfy the following items simultaneously: Women were between the ages of 20 to 50 years old with a history of sexual intercourse: Women were diagnosed with uncomplicated VVC or healthy vaginal microecology, had regular menstruation, and had been menstruating for at least 72 hours. Prior to this enrollment, there should be no vaginal irrigation and intravaginal administration for 1 week and no sexual intercourse for 72 hours. Exclusion criteria included: during the period of menstruation, pregnancy or lactation; a history of uterine or adnexal surgery; an incomplete medical record. Meanwhile, eligible women with normal vaginal microbiology were recruited as controls. Each participant volunteered to participate in this study and gave written informed consent. The study protocol was strictly performed with approval of the ethics committee of the First Affiliated Hospital of Xi’an Jiaotong University.

Women with uncomplicated VVC were treated with a single 500mg dose of Clotrimazole Vaginal Tablet plus a 7-day course of Lacidophilin Vaginal Capsules (2 tablets per day). The doses of probiotics administered ranged from ≥ 10 ^7^ CFU/day to 2.50 × 10^10^ CFU/day. In the present study, the dose was 1.20 × 10^7^ CFU/day. None of the patients developed complicated, recurrent, or severe VVC. Metronidazole (400mg, orally, twice a day) was given for 7 days during the follow-up period when BV was confirmed.

To ensure the rigor of the study, a healthy control group was designed in which the women had normal vaginal flora. More importantly, this study was also analyzed using a self-controlled observational method. To characterize the vaginal flora of VVC patients, we prospectively collected vaginal swabs from women with VVC without complications. The vaginal microbiota of healthy controls was sampled only at the first visit. In contrast, patients needed to be sampled 4 times consecutively, at time points m0 (first visit), m1 (8–10 days after the first visit), m2 (30 days after the second visit) and m3 (30 days after the third visit).

### 2.2. Sample collection

The patient was placed in the cystolithic position, and 3 vaginal swabs were placed on the lateral wall of the vagina. The first swab was stored in PBS and swiftly frozen in liquid nitrogen for at least 30 minutes. It was then transported to the laboratory and stored at -80°C for future use in the extraction of genomic DNA. The remaining 2 swabs were examined by expert laboratory technicians within 30 minutes in order to assess morphological and functional changes by microscopic examination. And these samples were collected by the same investigator and evaluated by the same 2 examiners. We would resort to a third veteran for the final diagnosis if the 2 inspectors were inconsistent in the assessment. The entire process followed standard biosafety and institutional safety procedures.

### 2.3. Diagnostic criteria

A normal vaginal microflora possesses the following characteristics: The vaginal pH was in the range of 3.8 to 4.5, the density and diversity of the vaginal flora was graded as II to III with the dominant flora being *Lactobacillus*, and the leukocyte esterase was negative. Combined with pelvic examination and laboratory tests, a diagnosis of VVC is made. Symptoms of thick white vaginal discharge, combined with the signs of edema and erythema, suggest the possibility of VVC. In addition, the combined findings of vaginal pH not exceeding 4.5, positive Gram stain, and the presence of budding or pseudobudding on oil microscopy further confirm the presence of VVC. BV was diagnosed and scored using the Nugent scale, and a score of 7 to 10 points was considered to be indicative of BV.

### 2.4. Genomic DNA extraction and 16S rRNA sequencing

The secretions were centrifuged at 10,000 g for 10 minutes. Genomic DNA was extracted using the QIAamp DNA Mini Kit (QIAGEN, Hilden, Germany) according to the manufacturer’s instructions. Finally, DNA was eluted with 100 *μ*L of buffer AE. The integrity and size of the extracted DNA was checked by electrophoresis with 0.5 mg/mL ethidium bromide on a 1% agarose gels. The mass and concentration of the DNA was determined (Synergy H1 Multimode plate reader, BioTek, Vermont).

The bacterial species composition and abundance in the vaginal communities were determined using culture-independent methods. The V1-V3 hypervariable region of the 16S rRNA gene was amplified using an optimized primer set. The amplified products were pooled for Illumina GAIIx 100 bp paired-end sequencing. The sequences generated after sequencing were determined by BLAST search. All reads were classified at the genus level using the QIIME software package. The composition of each genus was estimated based on specific reads. Genera that were contained in at least 2 samples were considered to be present in these samples and accounted for at least 0.1%. Principal component analysis (PCA) of different samples was calculated according to the phylogenetic Bray-Curtis metric with weighted uniFrac.

### 2.5. Statistical analysis

Quantitative data were presented as mean ± S.E.M., using the 2-tailed Student’s *t* test. While enumeration data were presented as numbers and percentages using chi-square and exact probability methods. All analyses were performed using SPSS 18.0, and *P* < .05 was considered statistically significant. Comparisons of pathogenic bacteria loads between groups were calculated using the Mann-Whitney U-test. In PCA analysis, the variance along principal components 1 and 2 was plotted along the horizontal and vertical axes. Shannon’s diversity was calculated for each sample in QIIME. Statistics were performed and figures were generated in R (V3.1.1). The Wilcoxon rank-sum test was used with a bonferroni multiple test correction to compare *α* and *β* diversity. Two analysis populations were defined as follows: the intent-to-treat (ITT) population included all participants who received ≥ 1 follow-up test. The per-protocol (PP) population included all participants who met all inclusion/exclusion criteria and received all doses of study visits within an acceptable time window.

## 3. Results

### 3.1. Subjects’ demographics

**Figure 1.**
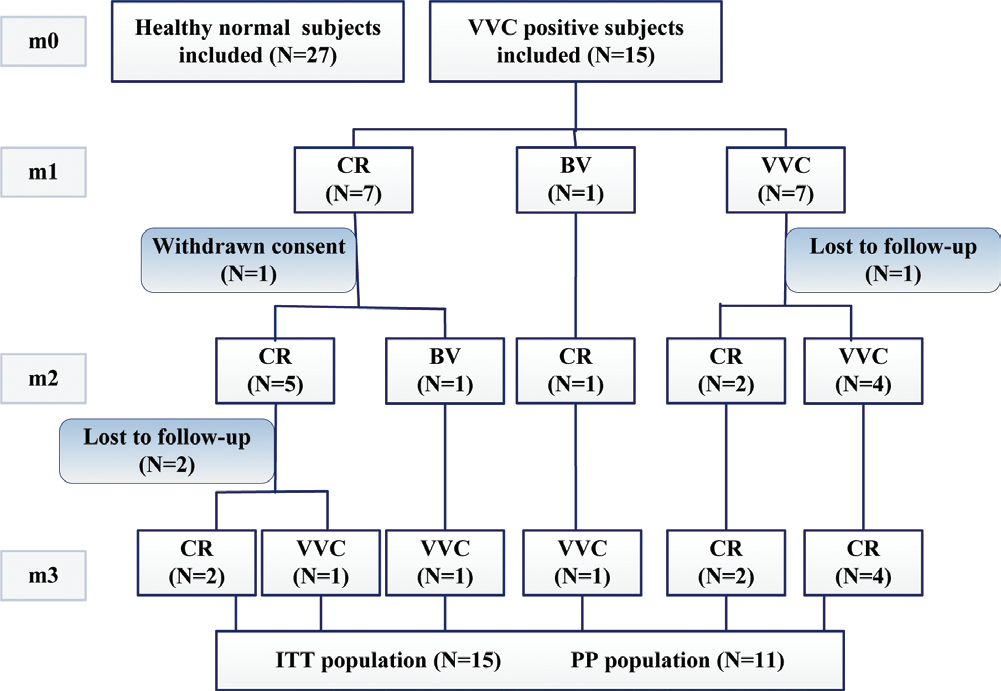
Study flow diagram. CR = complete remission, BV =bacterial vaginosis, VVC = vulvovaginal candidiasis, ITT = intent-intent, PP = per-protocol, m0 = the first visit, m1 = 8-10 days after the first visit, m2 = 30 days after the second visit, m3 = 30 days after the third visit, BV = bacterial vaginosis, VVC = vulvovaginal candidiasis.

**Figure 2.**
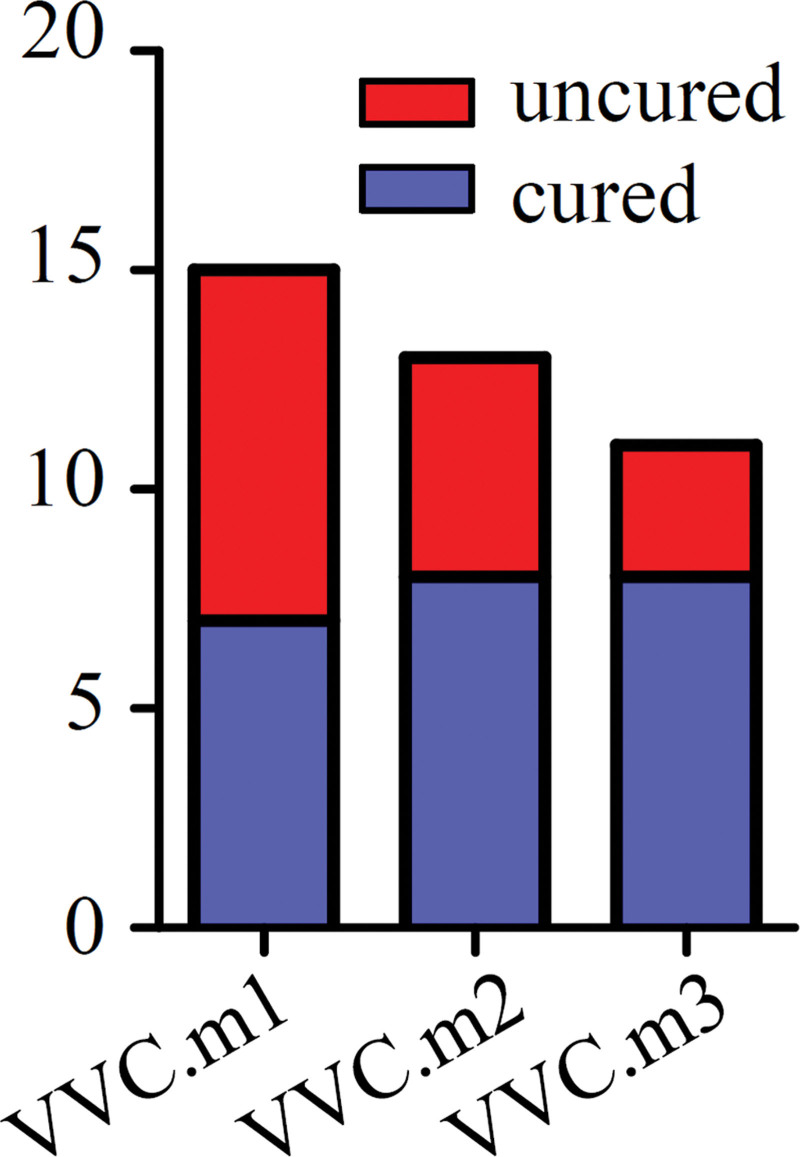
Treatment outcomes corresponding to clinical diagnosis. The red in the bar plot shows patients with failed treatment outcomes and blue shows successful treatment. Blue in the pie chart represents the rate of recurrence.

Twenty-seven women with normal vaginal flora and 15 women with uncomplicated VVC were enrolled in this study. The mean age in each group was 28.63 ± 5.40 years and 27.67 ± 3.33 years, with no statistical difference (*P* = .958). There was also no difference in the age at first sexual intercourse of women between the case group and the normal control group, with the mean age being 21.67 ± 0.58 years and 22.85 ± 0.46 years (*P* = .128). Baseline characteristics of participants are shown in Table 1. There were significant differences between the 2 groups in terms of contraceptive methods, history of vaginitis and history of HPV infection (*P* < .05). During the process of study, 1 patient withdrew the consent, 1 patient was lost to follow-up at the third visit, and 2 others dropped out at the last visit. At the end of visit, there were 3 recurring cases of VVC (Fig. 1). In total, 46.67% (7/15) of patients were cured at the second visit, 61.54% (8/13) at the third visit, and eventually 72.73% (8/11) in the PP population (Fig. 2).

**Table 1:** The baseline characteristics of participants.

Clinical parameters	Total	Case	Control	*P*
	(n = 42)	(n = 15)	(n = 27)	
Residence				
Rural area	9	2	7	.575*
Town and city	33	13	20	
Occupation				
Fixed work	30	12	18	.575*
Unemployment	12	3	9	
Educational background				
Secondary or below	11	2	9	.295*
College or above	31	13	18	
Menstrual phrase				
Luteal	21	8	13	.747
Follicular	21	7	14	
Reproductive history				
Multiparity	24	8	16	.710
Nullipara	18	7	11	
Dilatation and curettage				
Yes	20	7	13	.927
No	22	8	14	
Contraceptive methods				
Condom	25	6	19	.055
Others (ligation, IUD and so on)	17	9	8	
History of vaginitis				
Yes	14	9	5	**.006**
No	28	6	22	
History of HPV infection				
Yes	17	10	7	**.010**
No	25	5	20	

HPV = human papillomavirus, IUD = intrauterine device.

* Exact probabilistic method.

### 3.2. Sequencing results

**Figure 3.**
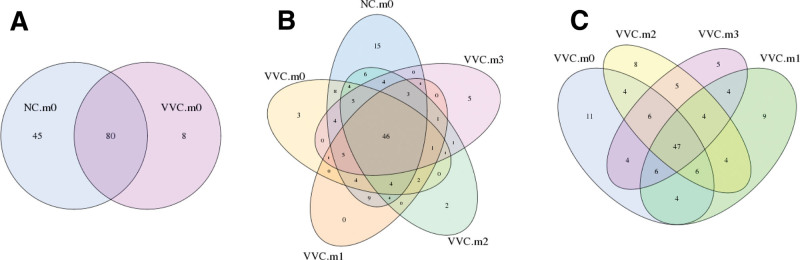
The OTU venn analysis. (A) the NC.m0 and VVC.m0 (B) the NC.m0 and VVC samples at different time points (C) VVC samples at different time points. Different color patterns represent different groups, the number of overlaps between different color patterns is the number of OTUs shared between 2 groups, and the number of overlaps between multiple color graphics is the number of OTUs between multiple groups. OTUs = operation taxonomy units, VVC = vulvovaginal candidiasis.

A total of 81 samples were sequenced, generating 1668 operation taxonomy units (OTUs) in all samples, with 653 OTUs in the normal control group and 1015 OTUs in patients’ samples. The OTU venn analysis showed there were 125 OTUs in the normal control group and 88 OTUs in patients’ samples, suggesting that the vaginal flora composition of healthy control women was relatively complex compared to that of VVC patients (Fig. 3A). As treatment progressed, the vaginal flora of VVC patients shared a greater large number of OTUs with the healthy individuals (NC.m0) (Fig. 3B). Subsequent follow-up visits (VVC.m1, VVC.m2 and VVC.m3) had more common OTUs compared to the vaginal microbiome in the VVC.m0 group (Fig. 3C). These data hinted that after the treatment with Clotrimazole plus Lacidophilin Vaginal Capsules, the vaginal microbiota of VVC patients tended to be consistent, similar to the normal vaginal flora.

**Figure 4.**
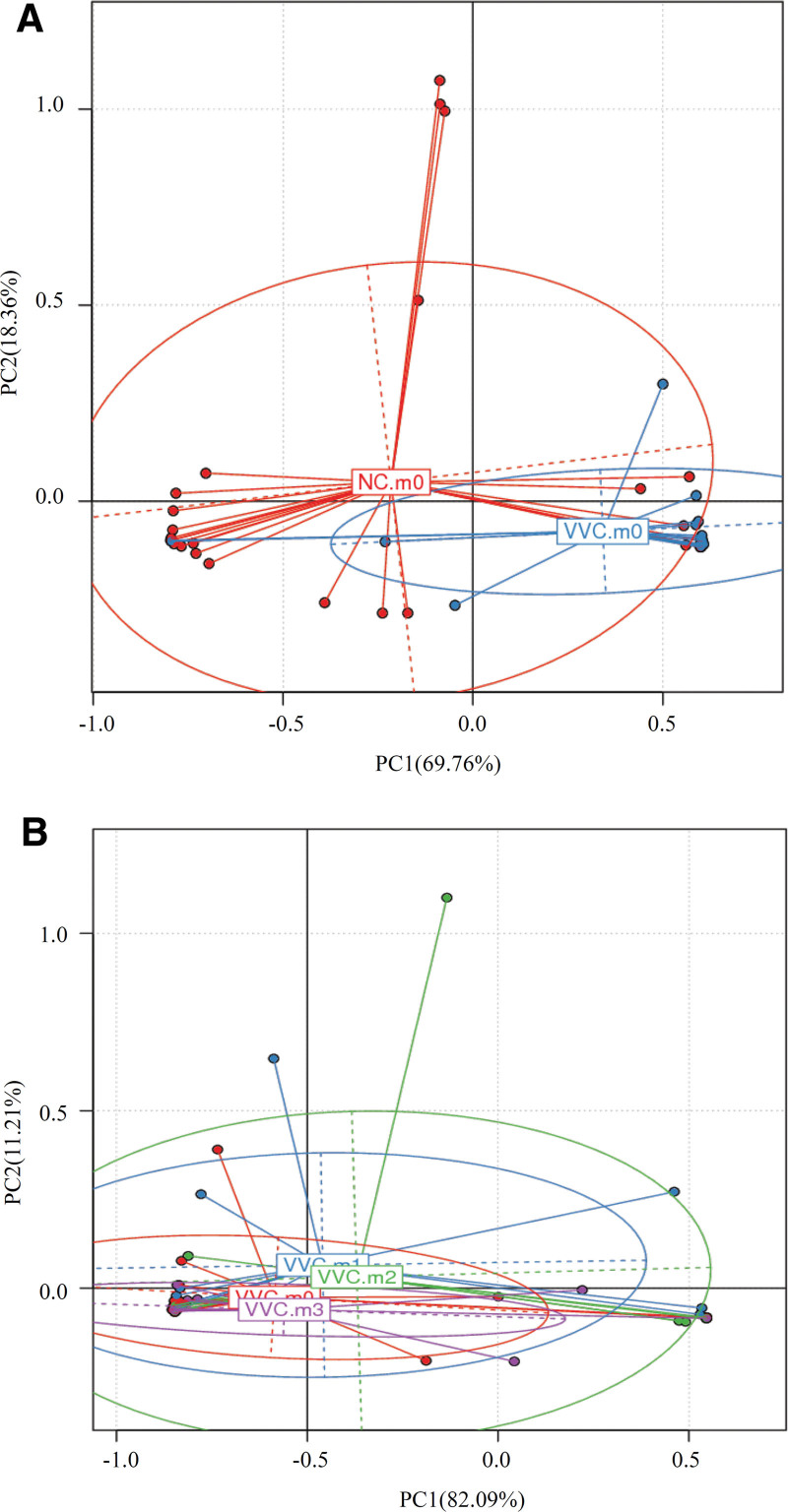
PCA analysis with weighted UniFrac distance. (A) the NC.m0 and VVC.m0 (B) VVC samples at different time points. The abscissa indicates the first principal component, the percentage in indicates the contribution of the first principal component to the sample difference; the ordinate indicates the second principal component, and the percentage indicates the contribution of the second principal component to the sample difference. The points in the figure indicate the respective samples. Different colors Representative samples belong to different groups. PCA = principal component analysis, VVC = vulvovaginal candidiasis.

PCA analysis showed that 2 samples (the NC.m0 group and the VVC.m0 group) were dispersed, while the samples from VVC.m1, VVC.m2 and VVC.m3 group became progressively closer (Fig. 4A, and B). These data indicated that there existed a great discrepancy between the vaginal microbiota of healthy normal individuals and cases. Encouragingly, the vaginal microbiome community structures tended to be similar after treatment with the combination of Clotrimazole and Lacidophilin Vaginal Capsules.

### 3.3. Microbiome diversity comparison

**Figure 5.**
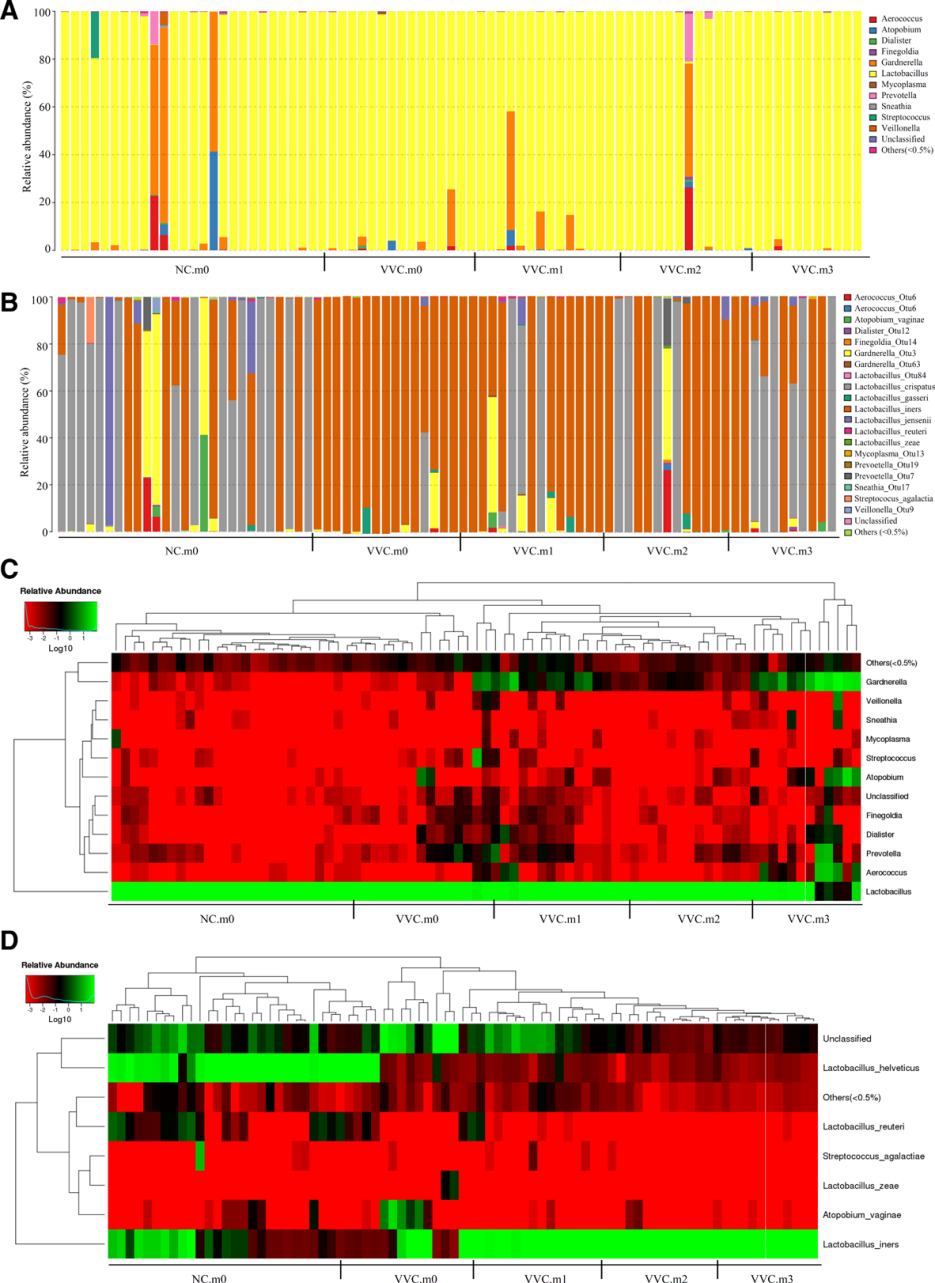
Vaginal microbiota composition of women in various groups. (A) at the Genus level (B) at the Species level (C) the heatmap graph based on the relative abundance of each species at the Genus level (D) the heatmap graph based on the relative abundance of each species at the Species level.

The bacterial composition of these vaginal communities in various groups at the Genus level is shown in Figure 5A. As shown, the dominant genus in each group was *Lactobacillus*, illustrating that there was no major difference between these groups. At the Species level, the bacterial composition of the women in the healthy control group was exceedingly abundant and dominated by *Lactobacillus*, especially *Lactobacillus. crispatus*, followed by *Lactobacillus. iners*, *Lactobacillus. jensenii* and *Gardneralla*. While the bacterial composition of women in the case group was relatively scarce and dominated by *Lactobacillus. iners* (Fig. 5B). These data suggest that the bacterial abundance of VVC patients gradually increased over the course of treatment. At the last visit, the abundance of vaginal flora was augmented further with the dominant bacteria being *Lactobacillus. crispatus*, followed by *Lactobacillus. iners* (Fig. 5B). The heat map plots based on the relative abundance of each species at the Genus level or Species level also showed the same change (Fig. 5C, and D).

**Figure 6.**
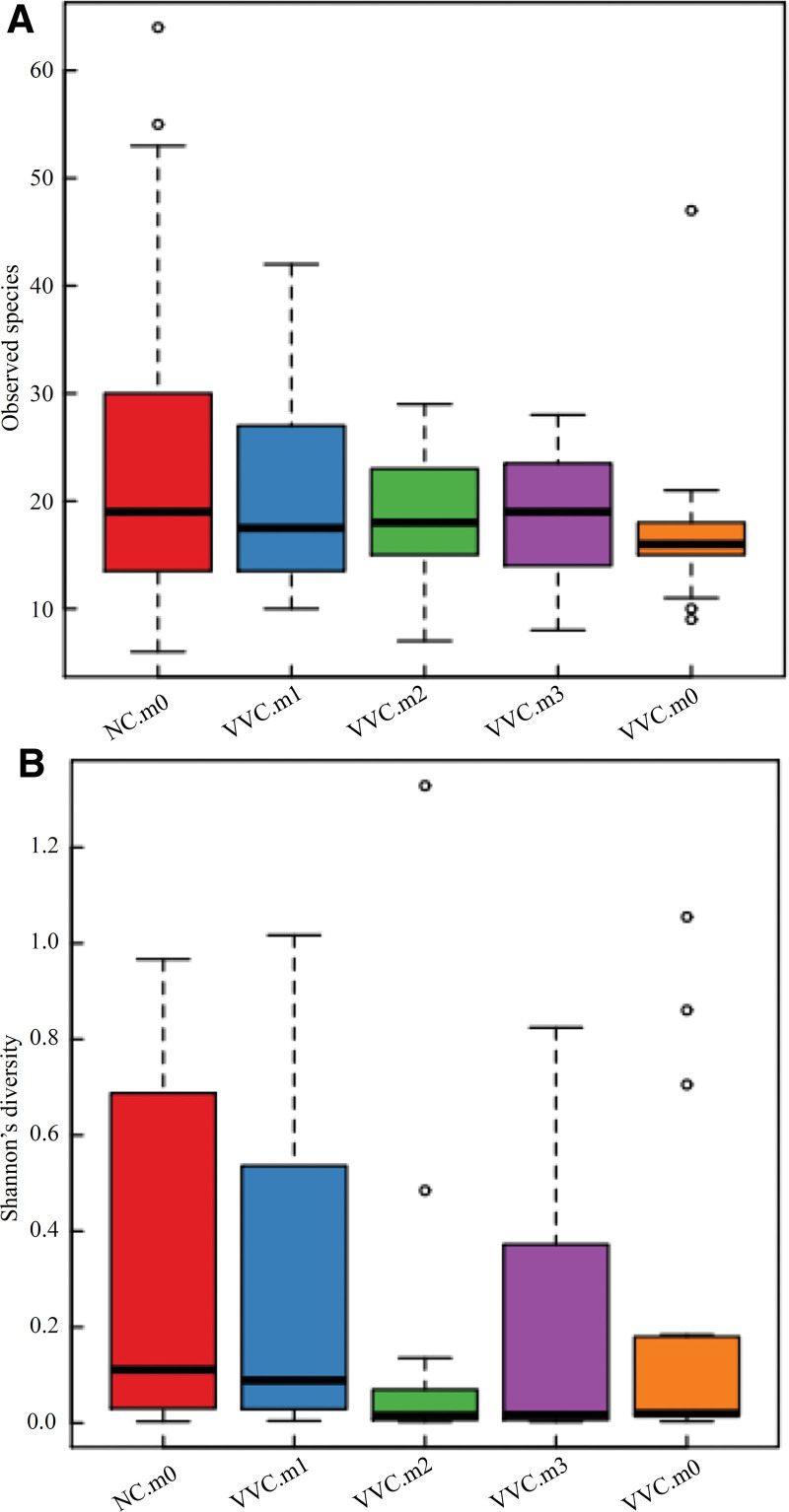
Distribution of *α* diversity within groups. (A) Diversity of bacterial communities in different groups at four time points (B) Shannon’s diversity; o: represents abnormal value.

After treatment, the bacterial diversity of VVC patients was increased gradually (Fig. 6A). At the same time, the Shannon index, which is a measure of the number of distinct microbes in a community, had increased significantly (Fig. 6B). These results altogether implied that the number of vaginal microbes was recovered after this combined treatment.

## 4. Discussion

VVC is the second most common cause of vaginitis, affecting about 75% of women of childbearing ages. Its repeated recurrence places a heavy burden on patients and poses a great threat to their quality of life. To make matters worse, drug resistance and the limited treatment options require innovative strategies for new and effective antifungal treatments. The vaginal microbiota in healthy women of childbearing ages is dominated by *Lactobacillus* species and is thought to enhance the defense against pathogenic microorganisms.^[17]^ Many studies suggest that disruption of the local microbial balance forms the basis of vaginal infections. Therefore, treatment should be based on correcting the dysbiosis by repositioning the initial resident *Lactobacillus* microbiota.

In recent years, the use of Lactobacilli-containing probiotic has emerged as a new strategy for the management of vaginal infections, improving vaginal microbial patterns by maintaining a normal vaginal lactobacilli microbiota.^[18,19]^ Various studies, in *vivo* and in *vitro*, have demonstrated that *Lactobacillus* can effectively inhibit the growth of *C. albicans* growth.^[20,21]^ Mechanisms of inhibition of *C. albicans* by *Lactobacillus* have been proposed, including competition with yeast for epithelial cell adhesion, inhibition of yeast growth by antibacterial products, and modulation of mucosal immune responses.^[22]^ In the present study, the combined cure rate for uncomplicated VVC increased with the course of treatment, reaching 72.73% at the final visit. Meanwhile, OTU venn analysis showed that the vaginal flora of VVC patients shared a greater number of OTUs with healthy individuals as treatment progressed. In addition, PCA analysis showed that the vaginal bacterial composition of VVC patients became progressively more tightly clustered. All these data suggested that the combined use of Clotrimazole Vaginal Tablets (500mg) and Lacidophilin Vaginal Capsules is effective in the treatment of uncomplicated VVC. The possible mechanism for this may be attributed to the improvement of the vaginal microbiota.

In order to determine which species of the vaginal microbiota should be obligatory for this infection, we compared and analyzed the microbiome diversity of the vaginal flora. The results showed that the dominant genus in each group was *Lactobacillus*. And the clear difference is that, at the Species level, the bacterial composition of the vaginal flora of healthy women was dominated by *Lactobacillus. crispatus*, while in patients with VVC, the predominant component was *Lactobacillus. iner*. These data suggested that, although the vaginal microbiota of women with VVC, especially the number of *Lactobacillus*, did not change too much, yet there happened a decisive change in the species of *Lactobacillus*. The change in dominant bacteria from *Lactobacillus. crispatus* to *Lactobacillus. iners* may be partly responsible for the occurrence of VVC. Two women who remained untreated throughout the course of treatment had a vaginal microbiome whose predominant component was *Lactobacillus. iners*. Therefore, we hypothesize that the composition of vaginal microbiome is an important factor in causing VVC, but not the only 1.

It is widely accepted that *Lactobacillus. crispatus*, an H_2_O_2_-producing strain, is the most common species in the vaginal microbiota of healthy women and contributes to the normal bacterial composition of the vaginal ecosystem. D-lactic acid has a more powerful protective effect against vaginal disease than the L- isomer by maintaining a relatively low pH in the vagina. Unfortunately, *Lactobacillus. iners* can only produce L-lactic acid and cannot synthesize D-isomer.^[23]^ D-lactic acid was at the highest level when *Lactobacillus. crispatus* was the dominant species and at the lowest level when *Lactobacillus. iners*, *Gardnerella or Streptococcus* were dominant in the vaginal flora. This phenomenon may explain the protective effect of *Lactobacillus. crispatus* against adverse reproductive outcomes not brought about by *Lactobacillus. iners*.^[24]^ Moreover, with the exception of *Lactobacillus. iners*, H_2_O_2_ produced by *Lactobacilli* not only inhibits the growth of pathogenic bacteria, but also prevents the invasion of exogenous bacteria. Thus, *Lactobacillus. iners*-dominant vaginal microbiota is usually associated with vaginal dysbiosis and appears to be less stable. In contrast, *Lactobacillus. crispatus*-dominant vaginal microbiota is usually associated with increased vaginal community stability and health. Therefore, changes in the structure and composition of the vaginal microbiota clearly affect the balance of the vaginal microecosystem and cause various vaginal infections.

## 5. Conclusion

This study suggests that Clotrimazole Vaginal Tablets plus probiotic Lacidophilin Vaginal Capsules could improve the efficacy of treating uncomplicated VVC and that this improvement in efficacy may be achieved by improving the composition of the vaginal flora and restoring vaginal microecology. However, the antifungal activity of probiotic *Lactobacillus* strains is very complex and requires further investigation. In addition, larger sample sizes and more stringent controls are warranted in future studies.

## Author contributions

Conceptualization: Xianling Zeng.

**Data curation:** Ruifang An.

**Formal analysis:** Xianling Zeng, Yafei Zhang.

**Funding acquisition:** Ruifang An.

**Methodology:** Xianling Zeng, Ruifang An, Yafei Zhang.

**Project administration:** Han Li.

**Resources:** Han Li.

**Software:** Han Li, Yafei Zhang.

**Validation:** Xianling Zeng, Yafei Zhang.

**Visualization:** Ruifang An.

**Writing – original draft:** Xianling Zeng.

**Writing – review & editing:** Xianling Zeng, Han Li.
